# A novel whole-team training programme for adult eating disorder services in England: rationale, development and preliminary evaluation

**DOI:** 10.1192/bjb.2024.20

**Published:** 2025-06

**Authors:** Kat Novogrudsky, Tom Gray, Emily Mitchell, Chris Attoe, Nikola Kern, Jess Griffiths, Lucy Serpell, Janet Treasure, Ulrike Schmidt

**Affiliations:** 1 King's College London, UK; 2 South London and Maudsley NHS Foundation Trust, London, UK; 3 University College London, UK

**Keywords:** Postgraduate medical education, foundation doctors, curriculum, competency, psychiatric placement

## Abstract

**Aims and method:**

In response to recommendations for improving the quality and coordination of care delivered by eating disorder services, a whole-team training programme was commissioned by Health Education England in 2020. This paper describes the development and evaluation of the Eating Disorder Services for Adults (EDSA) whole-team training course, delivered to National Health Service adult eating disorder community teams in England. Course participants (*n* = 561) in the first two EDSA training cohorts (2021 and 2022) were asked to complete questionnaires at intake and after each session, asking about their views on the training.

**Results:**

All course aspects were rated as highly enjoyable, meeting participants’ training needs and fostering reflective practice. Thematic analysis identified themes relating to key innovative features of the course and suggestions for improvements.

**Clinical implications:**

Preliminary evaluation suggests that EDSA is valued by clinicians to enhance their knowledge, skills and ability to improve eating disorder patient care.

Eating disorders are serious mental illnesses with high levels of mortality, disability and relapse, affecting up to 15% of females and 5% of males in England.^1^ Although there has been major government investment in child and adolescent eating disorder services,^2^ adult eating disorder services have not received parallel investment.^3^ In 2017, the Parliamentary and Health Service Ombudsman (PHSO) published a critical report^4^ highlighting shortcomings of eating disorder care in the National Health Service (NHS) and identifying several key areas for improvement, namely the need to improve the quality and availability of adult eating disorder services, better coordination within and transition between services, and improved training for health professionals. As part of the implementation strategy for the PHSO report, a commissioning guide for adult eating disorder services was published,^5^ together with a whole-team training (WTT) curriculum.^6^ Figure 1 shows the core principles of the curriculum. In 2020, a national training consortium led by clinical academics from King's College London and University College London was formed in response to a Health Education England (HEE) call for providers for WTT for adult eating disorder services.

**Fig. 1:**
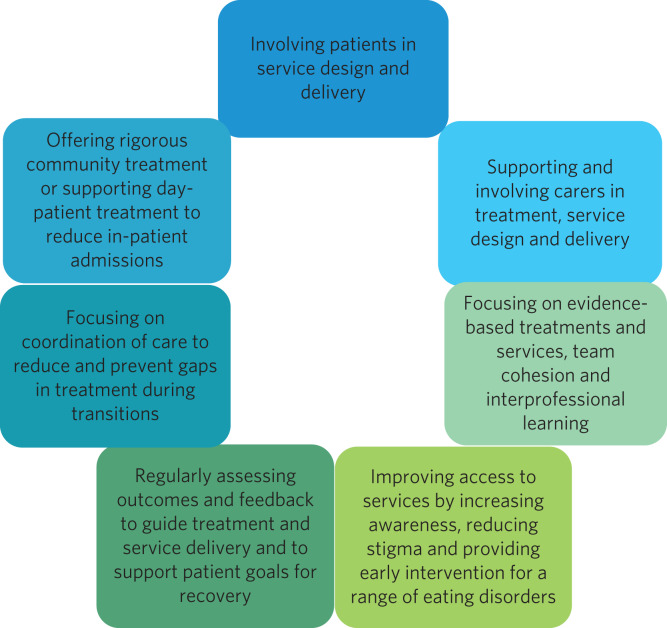
Core principles of the Eating Disorders Services for Adults (EDSA) curriculum.

Subsequently, the Eating Disorders Services for Adults (EDSA) WTT programme was developed under the auspices of Maudsley Learning, a mental health training and education provider, to address a range of challenges associated with adult eating disorder care in England. The aims of this paper are twofold: first, to describe the development of this unique training course, the thinking behind it, and its innovative features and content; and, second, to conduct a preliminary evaluation of the views and experiences of the first two cohorts of professionals undertaking this course.

## Method

### Development and content of the EDSA WTT course

The EDSA WTT course was developed by a multidisciplinary training consortium with expertise in eating disorders, together with lived experience experts (patients and carers), supported by Maudsley Learning. The group was mindful of the need to cover the substantial curriculum content which was distributed over eight full training days (Table 1). The curriculum was updated after each year in light of new evidence and to incorporate participant feedback.

**Table 1:** Broad course content, amalgamated from the 2 years delivered to date^a^

Day	Theme	Aims
1	Setting the scene	To provide an overview of the course and introduce mentor sessions, experiential exercises and team projects. To provide an overview of the societal, clinical and policy landscape in which adult eating disorder services are operating.
2	Assessment and treatment planning	To understand the tasks of assessment, engagement and formulation when caring for patients with eating disorders.
3	Medical management and comorbidities	To cover the physical and medical management of eating disorders, common comorbidities with eating disorders and their management (e.g. diabetes), and communication with patients and families about risk.
4	Nutritional management	To explore and discuss the roles of nutrition and diet in managing eating disorders, in addition to cultural diversity and its nutritional implications, and managing gut health.
5	Psychological treatments	To present what every clinician needs to know about evidence-based treatments for adult eating disorders and the patient experience of receiving these treatments, and to enable professionals to support the patient in choosing a treatment.
6	Overview of other treatments	To outline treatments and models of care and support beyond first-line therapies.
7	Patients who we struggle to help	To inform participants about patients with severe and enduring eating disorders, including those with comorbidities.
8	Innovative practice	To cover emerging treatments and novel service models with feasibility or pilot evidence.

a. The course content is updated annually to ensure relevance and responsivity to new challenges (e.g. workforce and waiting lists). Trainees have access to the content of all years. Each course day also had a mentor group session. These are not shown in the table.

Although it had originally been planned that course delivery would be face-to-face, the Covid-19 pandemic necessitated delivery entirely online. Several challenges emerged, including ensuring that the delivery format remained interactive, engaging and reflective. Further, course participants were from different disciplines and of different levels of seniority. Several features were incorporated to manage these challenges, including brief expert talks interspersed with lived experience sessions, and ample time for questions, feedback and discussion with experts and other participants in small mentor group settings. A virtual learning hub was created where course participants could revisit all past and current content.

A novel feature of the course was the strong lived experience component – this term is used to describe patients who have experienced eating disorder treatment and carers who have supported someone through eating disorder treatment. Lived experience experts were involved in all aspects of course development and delivery.

To deepen learning, all participating teams were allocated to a mentor group alongside one or two other teams, for collaborative within-team and across-team reflection. Mentors were experienced senior clinicians with supervisory expertise who were independent of their mentees. Prior to and during their role, mentors attended preparation and support sessions with the project team.

To ensure application of learning to everyday practice, each participating team was asked to undertake a quality improvement project relevant to one of the seven core principles of the training curriculum (Fig. 1). These projects were presented to the whole course cohort and further discussed in the mentor groups.

### Course participants

Course participants were NHS professionals (*N* = 561) working with adults with eating disorders in community settings. HEE managed course advertisement and recruitment, ensuring that access to training was distributed equitably across NHS regions in England. Although the course was designed as WTT, most teams sent a subset of clinicians on the training, meaning that these clinicians could work together as team representatives to bring tangible benefits back to their teams. At the end of the course, participants were provided with a certificate of attendance if they successfully completed at least 7 days of training.

### Course evaluation and analysis

End-of-day surveys were completed via SurveyMonkey and enquired about participants’ demographic information and professions (day 1 only), as well as their experiences completing the training. Participants rated their agreement with statements (e.g. overall, I enjoyed this training day) on a five-point scale from strongly disagree to strongly agree (with higher numbers indicating stronger agreement), in addition to rating certain aspects of the course (e.g. the speakers’ knowledge of the subject) on a five-point scale from inadequate to excellent (with higher numbers indicating better ratings). Free-text responses were also obtained (e.g. what will you do differently in the workplace after having participated in this course?).

Responses were downloaded and imported into IBM SPSS 28. Data were cleaned, and responses to scale-type questions were transformed into numerical values from 1 to 5, with higher values indicating more favourable responses (i.e. strongly disagree = 1, strongly agree = 5; inadequate = 1, excellent = 5).

Free-text responses were imported into NVivo and analysed following guidance from Braun and Clarke.^7^ After the main author had familiarised herself with the data, initial codes were identified, then combined into themes and subthemes. These were reviewed and defined, then reviewed again by co-authors.

### Ethics statement

As data had already been collected at the time this research was conducted, ethical approval was not obtained. All participants were aware that their data could potentially be used in future research.

## Results

### Respondents

**Fig. 2:**
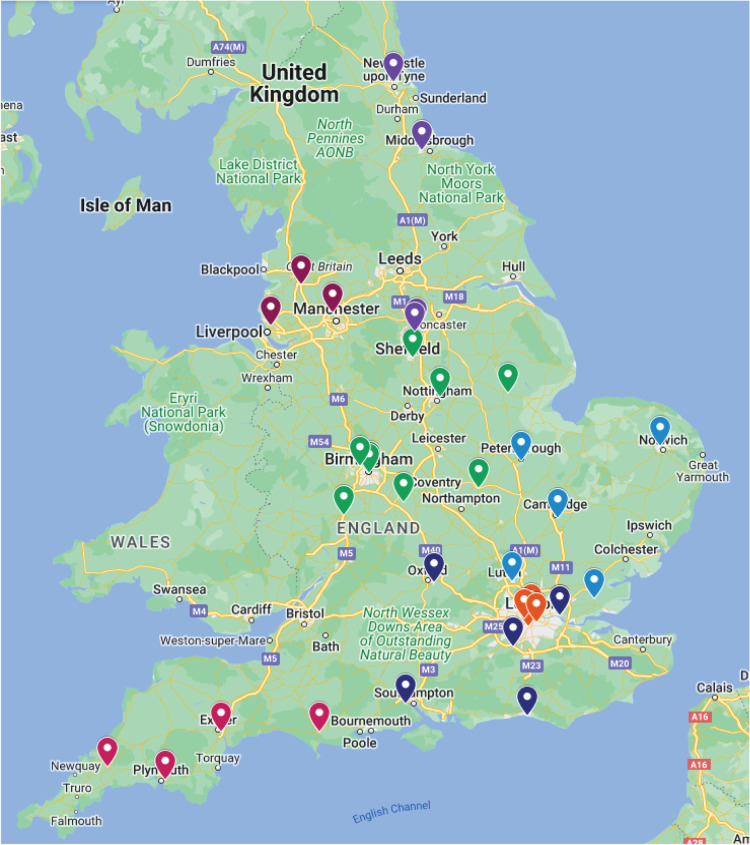
Map of National Health Service (NHS) trusts in which Eating Disorders Services for Adults (EDSA) participants were employed. Indicator colours correspond to NHS regions.

The 2021 EDSA training cohort consisted of 31 teams of eating disorder specialists, with 176 of the 276 (68%) registered delegates completing at least 7 days of training (Table 2). The 2022 cohort consisted of 36 teams of specialists, and 235 of the 285 (82%) registered delegates completed training (Fig. 2 and Table 3). Response rates were low, as survey items were optional. The term ‘respondent’ refers to those who responded to the survey, whereas ‘course participant’ refers to everyone that attended the training.

**Table 2:** Attendance rates and percentage of total registered delegates who attended each training day for 2021 and 2022 cohorts

	2021	Percentage	2022	Percentage
Total registered delegates	276	100	285	100
Day 1	249	90.2	261	91.6
Day 2	244	88.4	251	88.1
Day 3	225	81.5	243	85.3
Day 4	220	79.7	244	85.6
Day 5	194	70.3	240	84.2
Day 6	195	70.7	235	82.5
Day 7	195	70.7	219	76.8
Day 8	196	71.0	189	66.3
Number of certified delegates	176	63.8	235	82.5

**Table 3:** Demographic information for course participants

Characteristic	2021	2022
Age range, years		
20–24	2	4
25–29	10	14
30–34	2	24
35–45	13	28
46–55	16	22
>55	10	9
Not specified	1	
Gender		
Female	41	91
Male	3	9
Prefer not to say	1	1
Total	**45**	**101**
Ethnicity		
Black African/British	3	3
White British/Other White Background	36	98
Mixed	1	2
Indian/South Asian	2	3
Not specified	3	
Total	**45**	**106**
Profession		
Psychiatrist	5	7
Counselling psychologist	2	14
Clinical/practitioner psychologist	7	5
Assistant psychologist	7	4
Psychotherapist	4	11
Occupational therapist	7	9
Dietitian	8	14
General practitioner	1	2
Registered general nurse	1	0
Student nurse	0	1
Social worker	1	0
Support worker	3	4
Team manager	0	2

### Post-training day responses

Descriptive statistics were obtained for several questions regarding perceptions of the course. Ratings were consistently high across cohorts and throughout the training days, with all medians above 4. This indicates that respondents generally agreed with statements about the training being enjoyable and informative, and speakers and/or facilitators were rated positively (Table 4). End of survey responses were also largely positive, with all mean scores being above 80 out of 100 (Table 5).

**Table 4:** Median values and range of responses to feedback questions

	*N*	Enjoyment of the training	Did the training met your learning needs	Speakers/facilitators’ knowledge of the subject	Speakers/facilitators’ encouragement to participate and reflect	Speakers/facilitators’ enthusiasm
2021						
Day 1	40	4 (2,5)	4 (3,5)	5 (3,5)	4 (2,5)	5 (3,5)
Day 2	72	5 (1,5)	4 (1,5)	5 (3,5)	4 (2,5)	5 (3,5)
Day 3	49	5 (3,5)	5 (2,5)	5 (4,5)	5 (3,5)	5 (4,5)
Day 4	49	5 (2,5)	5 (2,5)	5 (4,5)	5 (3,5)	5 (4,5)
Day 5	25	5 (1,5)	4 (1,5)	5 (3,5)	5 (2,5)	5 (3,5)
Day 6	25	5 (1,5)	5 (1,5)	5 (3,5)	5 (2,5)	5 (3,5)
Day 7	121	5 (1,5)	5 (1,5)	5 (4,5)	5 (3,5)	5 (4,5)
Day 8	121	5 (2,5)	5 (3,5)	5 (4,5)	5 (3,5)	5 (4,5)
2022						
Day 1	167	5 (1,5)	5 (1,5)	5 (3,5)	5 (3,5)	5 (3,5)
Day 2	96	5 (1,5)	5 (1,5)	5 (2,5)	5 (2,5)	5 (3,5)
Day 3	96	5 (2,5)	5 (2,5)	5 (3,5)	5 (2,5)	5 (3,5)
Day 4	96	5 (2,5)	5 (2,5)	5 (3,5)	5 (2,5)	5 (3,5)
Day 5	110	5 (2,5)	5 (2,5)	5 (3,5)	5 (2,5)	5 (2,5)
Day 6	110	5 (1,5)	5 (1,5)	5 (3,5)	5 (2,5)	5 (2,5)
Day 7	45	5 (1,5)	5 (1,5)	5 (3,5)	5 (3,5)	5 (3,5)
Day 8	115	5 (2,5)	5 (2,5)	5 (4,5)	5 (2,5)	5 (3,5)

Numbers in parentheses are the range of responses to feedback questions. 1, unfavourable response; 5, favourable response.

**Table 5:** End-of-course survey responses

Cohort	*N*	Overall enjoyment of the training (%)	Overall, how valuable the training was for personal/career development (%)	Overall usefulness of the training for service development (%)	Percentage of participants who would recommend the course
		Mean [s.d.]	Mean [s.d.]	Mean [s.d.]	
2021	121	86.0 [19.5]	84.1 [21.6]	82.6 [21.4]	99
2022	115	85.6 [15.3]	83.9 [18.0]	83.8 [19.6]	98

0, unfavourable response; 100, favourable response.

### Qualitative analysis

**Fig. 3:**
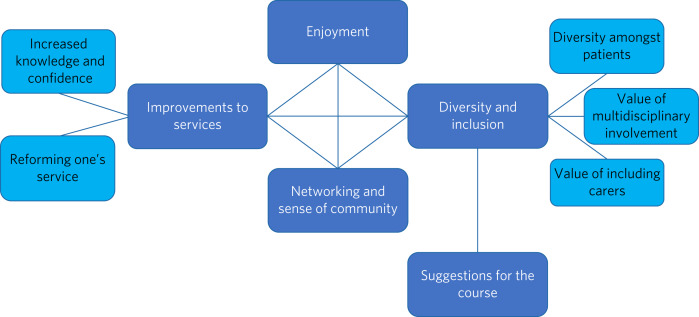
Map of qualitative themes.

Approximately 2600 comments were received and analysed. Five themes were identified: (a) overall enjoyment, (b) improvements to services, (c) commitment to focus on inclusion, (d) networking and communication, and (e) suggestions for improvements to the course. Although the exact number of respondents who agreed with each statement could not be determined owing to the high volume of comments, the subthemes were developed to reflect comments that were frequently repeated (Fig. 3).

### Theme: enjoyment

EDSA was generally well received by attendees, with several factors making the training especially enjoyable. These included speakers’ optimism and enthusiasm, lived experience input, and conversations on inclusivity and diversity. Most importantly, enjoyment came from respondents feeling that they had been ‘re-moralised’ and revitalised by the course, through being inspired by the content and feeling part of a community with shared experiences (Box 1).

Box 1 Quotes supporting themes and subthemes

Theme: enjoyment

Respondent (R)25: ‘The training far exceeded my expectations and was probably the most enjoyable training I have ever had the pleasure of being part of – this has further added to my passion for [eating disorders], and I eagerly await the next session’.

R27: ‘It has been an inspiring balance of education alongside provoking thought and emotion. I fully identified with feeling ‘morally injured’ as a clinician in our shadow pandemic. Today and looking ahead to future training days feels like a ‘soothing balm’ towards healing the ‘injury’ individually and systemically’.

Theme: improvement to services

*Subtheme: gained knowledge and confidence*

R1: ‘Greater awareness of medical risk will mean I am more confident and comfortable following up with this’.

R3: ‘As someone with very little experience of eating disorders, it was really helpful to get to some of the interventions and understand what can help and some of the issues’.

*Subtheme: reforming one's service*

R6: ‘Currently in the process of reviewing our waiting list protocol and how we support those waiting for treatment. Tomorrow, I plan to feedback today's content to inform decisions and changes to the service to hopefully roll out sooner rather than later’.

R11: ‘Found the whole day inspiring and will consider looking at improved monitoring of outcome measures and not be afraid to change approach if there is minimal change after a few weeks. Presentation and discussions around peer support and lived experience practitioners has provided much to reflect and ponder and consider ways service can be improved with incorporation of these possibilities’.

Theme: diversity and inclusion

*Subtheme: diversity among patients*

R7: ‘[I enjoyed] the feeling of a shared learning environment, optimism, and expert knowledge. I really enjoyed the inclusivity talk – and this will definitely change my practice’.

*Subtheme: value of including carers*

R10: ‘[Take-home message] Awareness of peer support/lived experience in service provision. Carer/family input and need for this to be embedded in outpatient services’.

*Subtheme: value of multidisciplinary involvement*

R12: ‘As an Occupational Therapist it was great to finally have a conversation about the value of [occupational therapy] within eating disorder services’.

R13: ‘I wish this training or similar [eating disorder] training is more widely available to student Dietitians because there isn't enough interest in the world of Dietetics in terms of recruiting into mental health for services to run effectively’.

*Subtheme: expansion of diversity, inclusivity and multidisciplinary discussions*

R14: ‘I also really enjoyed the session about inclusivity and cultural competence, BUT think that this session should be extended, with us taking time to consider other elements of diversity and inclusion’.

Theme: networking and sense of community

R17: ‘[I will] be more compassionate to myself, feel like we are doing badly, but actually across the country we are all struggling with the same issues’.

R18: ‘[I enjoyed] feeling part of a bigger picture – good to have national connections when we are the only eating disorders team in our trust’.

Theme: suggestions for the course

R21: ‘Some of the language was a bit technical at times. As we are all from different professions, the use of certain terms and acronyms didn't make sense…’.

R20: ‘These talks are aimed at people with no prior knowledge of eating disorders, and therefore my whole team found them quite condescending. I would have liked in-depth advice about how to manage type 1 diabetes and [eating disorders], rather than a patient's perspective on it…’.

R23: ‘It felt like listening to service users bashing services. We know our services are inadequate. I don't want to leave my incredibly busy job for the day to listen to service users complaining about how badly services work together’.

### Theme: improvements to services

#### Subtheme: gained knowledge and confidence

Respondents reported that the course was very informative and increased their knowledge of eating disorders and their diverse presentations and comorbidities, and of different treatments and support networks for patients. Respondents also felt more confident assessing and managing risks of eating disorders and delivering interventions.

#### Subtheme: reforming one's service

Respondents named several improvements that they would like to implement in their service and their teams following course attendance. The most common responses were plans to implement changes in assessment and formulation, being more patient-focused and improving rapport. Respondents voiced their intentions to bring peer support workers (i.e. recovering or recovered eating disorder patients who support others in their recovery) and lived experience practitioners into their services.

### Theme: diversity and inclusion

In addition to feeling more knowledgeable and confident when working with eating disorder patients, respondents frequently noted that they would be more mindful of patients, supporters and carers from populations that are typically underserved in eating disorder treatment or research.

#### Subtheme: diversity among patients

EDSA called attention to the needs and experiences of patients from minoritised backgrounds (including ethnic and socioeconomic backgrounds), making efforts to recognise differences across cultures, beliefs and environments and understand how these affect presentations and treatments. Speakers also stressed the importance of understanding patients with comorbidities or presentations which are less commonly seen in eating disorder services, such as patients with autism or diabetes.

#### Subtheme: value of including carers

A central aim of EDSA was to emphasise the need to include carers in treatment and service delivery. As such, carers were key contributors to the content and delivery of the course. The inclusion of carers’ lived experience and addressing the needs of carers was widely recognised and positively received by respondents.

#### Subtheme: value of multidisciplinary involvement

Eating disorder treatment requires the collaboration of multidisciplinary professionals. Improving understanding of what different professions have to offer may be a step towards improving service provision. Respondents appreciated the inclusion of professionals who are not as frequently mentioned in discussions around eating disorders, such as dieticians and occupational therapists.

#### Subtheme: expansion of diversity, inclusivity and multidisciplinary discussions

More information was frequently requested, with respondents noting that they would have appreciated additional conversations on the needs and experiences of patients from minority groups. Similarly, requests were made to include more information on occupational therapy and dietetics. Although this is important, it may not be feasible to incorporate additional content to the extent requested, owing to time constraints.

### Theme: networking and sense of community

A frequent response about what respondents enjoyed and would like more of was appreciation of the opportunity to network with other attendees. Some respondents indicated that participation in EDSA gave them a sense of community and a new perspective on the struggles experienced within their own team.

### Theme: suggestions for the course

Technical issues were frequently mentioned. In addition, respondents requested minor changes to the structure of the daily course schedule (e.g. more screen breaks). Respondents also requested additional opportunities for discussion and reflection. Some respondents felt that parts of the content were somewhat too advanced for their level. By contrast, some felt that the course content was pitched beneath their level of knowledge and expertise. Respondent 20 was particularly critical of this. Importantly, a minority of respondents felt disheartened and criticised by some of the conversations about service limitations and asked for them to be reframed and focused on potential solutions. However, such opinions were not common, with most respondents feeling empowered rather than criticised.

## Discussion

This paper describes and evaluates EDSA, a unique programme delivered to professionals working with adult eating disorder patients in NHS England. EDSA is the first programme of its kind with an integrated evaluation. Previously, a WTT for child and adolescent eating disorders services was conducted in England and was comparable with EDSA in length and format. However, this training, described by Eisler et al^2^, was only a one-off initiative and there was no evaluation included.

Eating disorders are challenging to treat and have heterogeneous presentations, and research on aetiological factors and treatments is constantly developing. In addition, the policy context in the NHS is constantly changing. Thus, it is important that training for eating disorder services is comprehensive, reflects the most up-to-date literature and allows learners to integrate new information into their practice.

EDSA is designed to support community adult eating disorder teams and create a supportive and inclusive learning environment committed to improving services across the nation, with further support provided by the rolling nature of the programme. The overall positive feedback suggests that the course aim of creating flourishing, learning teams was achieved. Survey results suggest that the reception of the course was overwhelmingly positive, with consistently high ratings throughout the training days and across the two cohorts. By the end of training, a majority of survey respondents (cohort 1 = 99%, cohort 2 = 98%) indicated that they would recommend EDSA to other professionals. These findings were reinforced by qualitative responses. Thematic analysis identified five themes and several subthemes. These are discussed below.

Attendees felt that several factors made the training especially enjoyable, including speakers’ optimism and enthusiasm, input from trainers with lived experience, peer support, and conversations on inclusivity and diversity.

Respondents felt that the course content was relevant, nuanced and met their learning needs. The majority reported perceived increases in their knowledge and confidence when working with patients with eating disorders. Respondents also mentioned their intentions to implement their newfound knowledge in their practice and relay their learning to colleagues.

Common responses included appreciation for the course's commitment to diversity and inclusion and underserved groups. For example, the course included discussion about the needs and experiences of patients from minoritised backgrounds, considering how various cultures, beliefs and environments may influence eating disorder presentations and subsequent treatments. In this context, the need for an intersectional approach to assessment and treatment was recognised, as, for instance, culturally informed interventions produce more favourable outcomes for patients than non-intersectional approaches.^8,9^

Conversations surrounding underserved populations with complex needs, such as those with comorbid autism spectrum disorder or type 1 diabetes mellitus were also felt to be useful, as these conditions are less frequently included in eating disorder discourse.

Moreover, a central aim of EDSA was met by co-producing and co-delivering the course with trainers with lived experience as part of the training team, and this was well received by respondents. Similarly, the involvement of peer support workers in services was rated as thought-provoking and informative, with the expectation that these individuals would augment services – this aligns with literature outlining the benefits of lived experience for patient and clinician outcomes.^10^

Finally, respondents appreciated the inclusion of a range of professionals, such as dietitians and occupational therapists, in conversations about the contributions of the multidisciplinary team, suggesting that a broad representation is needed. Discussing the unique contributions of these professions in an eating disorder context may motivate teams to consider greater interdisciplinary collaboration. Ultimately, this may enhance the patient experience.

The course allowed respondents to feel part of a larger community dedicated to the betterment of eating disorder patient care. Respondents also described a shared sense of struggle while trying to learn and support each other. Respondents appreciated the opportunity to network and requested more time to interact with attendees outside their teams. Obviously, a fully virtual course environment, as was delivered here, does not provide the same quality and depth of personal contact as would be provided in a face-to-face environment. In the future, hybrid forms of course delivery may therefore be desirable.

Course feedback pointed towards areas for improvement. Some respondents felt that the course content was pitched at too high or low a level for them, which was unsurprising, given the diversity of professional backgrounds and levels of seniority among course participants. The virtual resources provided aimed to ameliorate this issue, although more information on some topics was still requested. Future deliveries could provide these extra materials, including more beginner-level summaries and terms, as well as more advanced in-depth information to support all levels of learning. Some respondents also suggested adding additional interactive components, such as optional quizzes.

A small number of respondents expressed feeling disheartened and judged in response to criticism of the NHS. The aim of the course is to motivate professionals and provide them with opportunities to share learning, problem-solve and create action plans to ameliorate the challenges faced across the NHS. It is important that speakers and mentors remain sensitive to these issues and emphasise that no one individual, team or trust is responsible for systemic shortcomings in service provision.

### Limitations

Low survey completion rates limited this research and may suggest selection bias in the responses received. However, it is likely that inconsistencies in response rates were due to individual clinicians’ work demands and a lack of time to provide detailed feedback. Attendance rates also declined across training days. Similarly, this may have been owing to work demands, rather than interest in the content on each day or engagement with the training. Fluctuations in response rates across cohorts were evident. These could have been the result of slight changes in course content; however, they were possibly due to insistence from facilitators for participants to complete surveys. Pre- and post-training changes in knowledge could not be compared, as these measures were not included in the surveys. The lack of standardised questionnaires to assess training outcomes was another limitation. The wide standard deviations suggest that answers were quite variable; hence, mean values must be interpreted with discretion. Last, there was no longitudinal follow-up for this study, although this limitation is being addressed in further investigations.

### Implications for research and practice

Few studies on effective team training have been conducted in mental health settings. Longer-term effects of WTT on participants and the care they deliver are also seldom investigated. Further research is needed to confirm the reported benefits of training interventions in mental healthcare in general and for eating disorders specifically. A larger study is currently underway.

This training has the capacity to improve patient outcomes and represents value for money owing to the increased coordination of care, more awareness and confidence from first-contact staff to expedite identification of patients, delivery of pre-treatment interventions and provision of support for those on waitlists, support for and involvement of carers, and provision of the most effective and cost-effective evidence-based treatments. The online format of EDSA provides an advantage in terms of cost and reach across community services. The at-home training reduces costs for travel, accommodation and catering and circumvents issues pertaining to travel disruptions, e.g. from train strikes. This also allows the training to reach participants who may be unable to travel far to attend in-person sessions.

Currently, EDSA is only available for NHS clinicians in England; however, other UK jurisdictions (e.g. Scotland) have shown interest in delivering EDSA to their teams, and there has also been interest from international clinicians and teams keen to attend the training. With careful cultural, region-specific and healthcare-system-specific adaptations for different settings and countries, EDSA has the potential to spread its success internationally. As a result of the first two successful deliveries, the HEE has extended the programme until 2026, evidencing its value for staff and patients.

Overall, EDSA appears to be a beneficial training programme which is positively received by respondents. Attendance enhances feelings of confidence and supports learning when working with eating disorder patients and carers. EDSA also allows for the opportunity to network with other professionals and discuss important topics in the field of eating disorders and motivates clinicians to make changes in their services. Areas for improvement will be addressed in future deliveries, and further research might bridge some gaps in the literature on clinician needs and well-being when working with patients with eating disorders. Ultimately, however, even highly motivated and trained clinical teams can only deliver high-quality care to patients and families if the services they operate in are appropriately resourced. Much more needs to be done to achieve this.^3^

## Data Availability

The data that support the findings of this study are available from the corresponding author, K.N., upon reasonable request.

## About the authors

**Kat Novogrudsky** is a PhD candidate in the Department of Psychological Medicine, Institute of Psychiatry, Psychology & Neuroscience (IoPPN), Kings College London, UK. **Tom Gray** and **Emily Mitchell** are Programme Managers at Maudsley Learning, South London and Maudsley NHS Foundation Trust, London, UK. **Chris Attoe** is the Head of Research & Development at Maudsley Learning, South London & Maudsley NHS Foundation Trust, London, UK. **Nikola Kern** is a Consultant Psychiatrist in the South London and Maudsley NHS Foundation Trust, London, UK. **Jess Griffiths** is a National Co-Lead for First episode and Rapid Early intervention for Eating Disorders (FREED), Deputy Curriculum Lead for Eating Disorders Services for Adults (EDSA), Eating Disorders Therapist and Lived Experience Lead at Maudsley Learning, South London and Maudsley NHS Foundation Trust, London, UK. **Lucy Serpell** is Professor of the Psychology of Eating Disorders at University College London, UK. **Janet Treasure** is Professor of Adult Psychiatry in the Department of Psychological Medicine, IoPPN, Kings College London, UK. **Ulrike Schmidt** is Professor of Eating Disorders in the Department of Psychological Medicine, IoPPN, Kings College London, UK.
